# Déficit congénital sévère en facteur VII découvert fortuitement en péri-partum: à propos d’un cas

**DOI:** 10.11604/pamj.2025.51.15.47005

**Published:** 2025-05-14

**Authors:** Youssef Aarjouni, Oumayma Elbouni, Chikhi Brahim, Mohamed Benani, Houmed Housein, Laila Drouzi, Nezha Oudghiri, Sofia Lahbabi, Rajaa Tachinante

**Affiliations:** 1 Service d'Anesthésie Réanimation, Hôpital Militaire d'Instruction Mohammed V, Université Mohammed V, Rabat, Maroc,; 2 Service d'Hématologie Biologique, Hôpital Ibn Sina, Université Mohammed V, Rabat, Maroc,; 3 Service d'Anesthésie Réanimation Obstétricale, Hôpital de Maternité Souissi, Université Mohammed V, Rabat, Maroc

**Keywords:** Déficit en facteur VII, coagulation, hémorragie péripartum, césarienne, cas clinique, Factor VII deficiency, coagulation, peripartum haemorrhage, cesarean section, case report

## Abstract

Le facteur VII, ou proconvertine, est une protéine de la coagulation dépendante de la vitamine K, intervenant dans la voie extrinsèque de la coagulation. Son déficit congénital constitue une affection rare à transmission autosomique récessive et peut se manifester par des épisodes hémorragiques de sévérité variable. Nous rapportons le cas d´une patiente de 22 ans, primigeste, chez qui un trouble de la coagulation a été découvert de manière fortuite en péripartum à la suite d´un épisode d´épistaxis. Le bilan biologique a révélé un effondrement du temps de prothrombine ainsi qu´un allongement significatif de l'International Normalised Ratio (INR), conduisant au diagnostic d´un déficit sévère en facteur VII. Compte tenu du risque hémorragique, une prise en charge obstétricale spécifique a été mise en place. L´accouchement par césarienne a été réalisé sous anesthésie générale, après une préparation préopératoire comprenant une transfusion de plasma frais congelé et l'administration d´un antifibrinolytique. L'intervention s´est déroulée sans complications, et l'évolution post-opératoire a été favorable, sans épisode hémorragique notable. À travers cette observation, nous mettons en lumière l'importance du dépistage précoce et d´une prise en charge adaptée du déficit en facteur VII dans un contexte obstétrical, afin de minimiser les risques hémorragiques tant pour la mère que pour le nouveau-né.

## Abstract (en)

Factor VII, also known as proconvertin, is a vitamin K-dependent coagulation protein involved in the extrinsic coagulation pathway. It is a congenital deficiency that is a rare autosomal recessive disorder that can manifest as bleeding episodes of varying severity. We here report the case of a 22-year-old primigravida patient in whom a coagulation disorder was incidentally discovered during the peripartum period, following an episode of epistaxis. Biological investigations revealed a significant decrease in prothrombin time and a markedly prolonged INR, leading to the diagnosis of severe factor VII deficiency. Given the high risk of hemorrhage, a specific obstetric management plan was implemented. Delivery was performed by cesarean section under general anesthesia, following preoperative preparation that included a transfusion of fresh frozen plasma and the administration of an antifibrinolytic agent. The procedure was uneventful, and the postoperative course was favorable, with no significant bleeding episodes. This study highlights the importance of early detection and appropriate management of factor VII deficiency in an obstetric context to minimize hemorrhagic risks for both mother and newborn.

## Introduction

Le déficit congénital en facteur VII est une maladie hémorragique rare à transmission autosomique récessive, dont la prévalence est estimée entre 1 sur 500 000 et 1 sur 1 000 000 naissances [1]. Ce trouble est caractérisé par une hétérogénéité clinique allant d´une absence de symptômes à des hémorragies sévères [2]. Le traitement est de type substitutif, par l'administration de concentré de facteur VII [3]. Nous rapportons ici le cas d'une primigeste de 22 ans chez qui ce déficit a été découvert fortuitement en période péripartum, nécessitant une prise en charge obstétricale et anesthésique adaptée afin de prévenir tout risque hémorragique maternel et néonatal.

## Patient et observation

**Informations relatives à la patiente:** il s'agit de madame S.A. âgée de 22 ans, G1P0, sans antécédents notables, pas de notion de consanguinité. La patiente a présenté un épisode d'épistaxis sans retentissement clinique une semaine avant son admission. Un bilan a été réalisé objectivant un trouble de coagulation de découverte fortuite, puis la patiente a été adressée à l´Hôpital de maternité Souissi de Rabat pour complément de prise en charge.

**Chronologie:** la patiente s´est présentée aux urgences obstétricales de l'Hôpital de maternité Souissi le 14 novembre 2024, grossesse estimée à 41 SA selon la date des dernières règles (DDR) précise et échographie de 11 SA, suivie en privé et adressée dans notre formation pour accouchement à risque suite à la découverte fortuite d'un bilan d´hémostase réalisée le 14 novembre revenu perturbé avec un taux de prothrombine (TP) à 23%, un temps de céphaline activée (TCA) normal à 1,2 et un *International Normalised Ratio (INR)* allongé à 5,8; un deuxième bilan réalisé le même jour dans un laboratoire d'analyses biologiques différent confirme les perturbations.

**Démarche diagnostique:** 14 novembre 2024: patiente admise à l'Hôpital de maternité Souissi pour anomalie de coagulation, le bilan de première intention réalisé objectivant un TP a 28% et TCA normal avec un *INR* allongé, la numération formule sanguine a objectivé une hémoglobine a 12g/dl, un hématocrite à 34,4%, des plaquettes à 104000 et un taux de fibrinogène normal. Le 15 novembre 2024: bilan de 2^e^ intention révélant un déficit assez profond du facteur VII avec une activité à 0,1% pour une normale entre (50% - 130%).

**Diagnostic:** déficit congénital rare du facteur VII découvert de façon fortuite en péri-partum.

**Résultats cliniques:** l'examen clinique et obstétrical de la patiente a objectivé une hauteur utérine à 32cm, des bruits cardiaques fœtaux positifs avec 2 contractions par 10 minutes, par ailleurs l'examen au speculum ne trouve pas de saignement. La scannopelvimétrie objective un pronostic d'accouchement par voie basse médiocre, avec un indice de Magnin à 20-21cm et un diamètre bi sciatique à 8cm, donc un accouchement par voie haute est préconisé.

**Interventions thérapeutiques:** la rachianesthésie étant contre-indiquée, l'accouchement par voie haute serait réalisé sous anesthésie générale. La préparation de la patiente passe par transfusion de plasma frais congelé 10ml/kg en préopératoire et d'anti fibrinolytique à savoir 1g exacyl durant l'accouchement. Par ailleurs, le monitorage fœtal par rythme cardiaque fœtal (RCF) a révélé des micro-oscillations avec une tachycardie fœtale d´où l´indication d´extraction urgente. Patiente admise au bloc le 15 novembre 2024 à 5h du matin, installation en décubitus latéral mise sous oxygène, monitorage standard non invasif, sondage vésical, badigeonnage par de la bétadine et mise en place des champs stériles; induction en séquence rapide par du xylocaïne 60mg + propofol 100mg + rocuronium 50mg, manœuvre du Sellick et intubation orotrachéale facile par une sonde 6,5. Après vérification de la bonne position de la sonde, gonflage du ballonnet et fixation de la sonde. Incision réalisée à 05h10, extraction d´un nouveau-né de sexe masculin, score Apgar à 10, poids à 4kg300, puis injection de 150ug de fentanyl. Le per-opératoire s'est passé sans incident, pas de saignement anormal, hémostase assurée, en fin d'intervention un drain pariétal a été mis en place.

**Suivi et résultats des interventions thérapeutiques:** les suites post-partum ont été simples pour la mère et le nouveau-né. Patiente admise à la réanimation obstétricale pour surveillance rapprochée vu le risque hémorragique élevé secondaire au trouble de la coagulation. Le bilan d´hémostase réalisée le 16 novembre objective un TP toujours bas à 21% et un *INR* allongé à 3,26 sans saignement clinique. Patiente transférée au service de médecine interne pour complément de prise en charge.

**Consentement éclairé:** la patiente est consentante pour la publication de son cas clinique après avoir été avisée de l'intérêt scientifique de partager sa pathologie rare.

## Discussion

**Figure 1:**
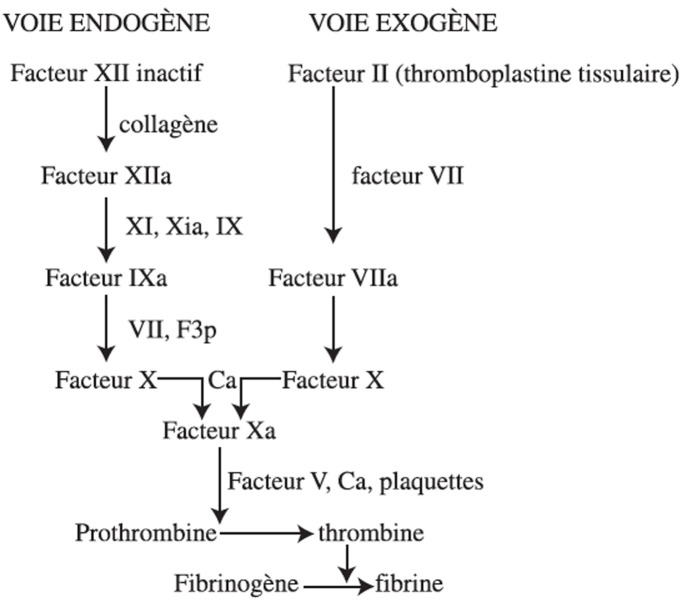
rappel physiologique

Le déficit en facteur VII de la coagulation a été décrit pour la première fois en 1951 par Alexander. Il s'agit d'une maladie rare, dont la prévalence est estimée entre 1 sur 500 000 et 1 sur 1 000 000 [4]. Toutefois, environ 1 personne sur 500 est porteuse du gène défectueux. Ce trouble est de transmission autosomique récessive, affectant ainsi aussi bien les hommes que les femmes. Le gène impliqué est situé sur le chromosome 13 [5]. Bien que rare, ce déficit est plus fréquent dans les populations où les mariages consanguins sont répandus, d´où l´importance de stratégies de dépistage permettant un diagnostic précoce [6]. Le facteur VII, également appelé proconvertine, est une glycoprotéine sanguine produite par le foie et impliquée dans la cascade de la coagulation. Une fois activé par la thromboplastine tissulaire, il joue un rôle clé en activant à son tour les facteurs X et IX, favorisant ainsi la poursuite du processus de coagulation (Figure 1) [7].

Le déficit en facteur VII se caractérise par une grande hétérogénéité génétique et clinique. Du point de vue génétique, le syndrome hémorragique s´observe chez les sujets homozygotes; un sujet hétérozygote n´en est que très rarement affecté [8]. Du point de vue symptomatique, l´âge d´apparition des symptômes est variable: plus le déficit est détecté tôt, plus les saignements sont spontanés et sévères. On distingue quatre formes cliniques de la maladie [2]: une forme grave à la naissance et la première enfance, caractérisée par l´hémorragie à la chute du cordon et par l´hémorragie intracrânienne; une forme grave avec début dès la deuxième enfance, caractérisée par une hémorragie de la chute des dents de lait ou à la puberté; une forme légère avec un début tardif parfois à l´âge adulte avec des saignements après un traumatisme ou après une intervention chirurgicale (le cas de notre patiente qui est restée asymptomatique jusqu´à l´âge adulte); une forme presque asymptomatique découverte lors d´une enquête génétique ou d´un bilan préopératoire.

Le déficit en facteur VII est suspecté en présence d´un temps de Quick allongé avec un temps de céphaline + Kaolin (TCK) normal. La confirmation diagnostic repose sur le dosage du facteur VII, dont les valeurs normales varient entre 70% et 140% [8]. L´enquête familiale est nécessaire, notamment le dosage du facteur VII chez les parents et la fratrie du sujet atteint.

Le traitement repose sur des mesures symptomatiques indiquées lors d´un saignement aigu, et consiste en [9]: l'administration de plasma frais congelé à une dose de 10 à 15ml/kg, avec certains inconvénients: petite concentration de facteur VII, nécessité de grand volume pour être efficace, et risque de transmission des virus; l´administration du concentré de complexe prothrombinique (PPSB) de moins en moins utilisé du fait du risque élevé de complications thromboemboliques; l´administration de concentré de facteur VII, en sachant que 0,5U/kg fait augmenter le taux de proconvertine de 1%.

Une prophylaxie péri-opératoire reste indiquée en [9]: préopératoire: 20U/kg afin d´atteindre un taux de 40%; et en postopératoire: 5 à 10 U/kg par huit heures pendant 5 à 10 jours en vue de maintenir un taux 20%.

Dans notre contexte, notre patiente a bénéficié d'une administration de PFC en préopératoire avec une bonne évolution et sans saignement anormal.

## Conclusion

Le déficit en facteur VII est une pathologie rare et souvent méconnue, dont la gravité clinique varie en fonction du taux résiduel de proconvertine. Son diagnostic repose sur l´allongement du temps de Quick avec un TCK normal, confirmé par le dosage spécifique du facteur VII. Dans un contexte obstétrical, ce déficit représente un défi majeur en raison du risque accru de complications hémorragiques maternelles et néonatales. La prise en charge repose principalement sur l'administration de plasma frais congelé (PFC), bien que son efficacité soit limitée par la faible concentration en facteur VII et le risque de surcharge volémique. L'utilisation de concentrés de complexe prothrombiniques (PPSB) est de plus en plus délaissée en raison du risque thromboembolique élevé. L'administration de facteur VII recombinant (rFVIIa) constitue actuellement l'option thérapeutique de choix, permettant une correction rapide du déficit avec un risque thrombotique moindre. Cependant, son coût élevé et sa disponibilité limitée en restreignent l'accès dans certains contextes. Le dépistage précoce du déficit en facteur VII, notamment lors d'un bilan préopératoire ou en cas d´antécédents familiaux, revêt une importance capitale. Il permet d'adapter la prise en charge anesthésique et obstétricale afin de prévenir les complications hémorragiques péripartum. Une surveillance post-partum rigoureuse est également essentielle, particulièrement chez les patientes ayant bénéficié d'un traitement substitutif, pour éviter toute récidive hémorragique secondaire. Enfin, ce cas illustre l'intérêt d'une approche multidisciplinaire impliquant obstétriciens, anesthésistes et hématologues afin d'optimiser la prise en charge des patientes présentant un trouble rare de la coagulation. L'amélioration des stratégies de dépistage et l'accessibilité aux traitements innovants restent des enjeux majeurs pour réduire la morbi-mortalité associée à cette affection.
